# Supplementary material for: Transcriptional Activation of Ecdysone-Responsive Genes Requires H3K27 Acetylation at Enhancers
Source: Int J Mol Sci. 2022 Sep 16;23(18):10791. doi: 10.3390/ijms231810791 (PMC9502983; doi:10.3390/ijms231810791)
Supplement: Supplementary file 1 [file ijms-23-10791-s001.zip › ijms-1879961-supplementary/ijms-1879961-supplementary.pdf]

Transcriptional activation of ecdysone-responsive genes requires H3K27 acetylation at enhancers

Dong Cheng[1,2], Zhaoming Dong[1,2], Ping Lin[1], Guanwang Shen[1,2] and Qingyou Xia[1,2,*]

[1] State Key Laboratory of Silkworm Genome Biology, Biological Science Research Center, Southwest University, Chongqing 400715, China

[2] Chongqing Key Laboratory of Sericultural Science, Southwest University, Chongqing 400715, China

* Corresponding author: Email: xiaqy@swu.edu.cn

***Supplementary figures*:**

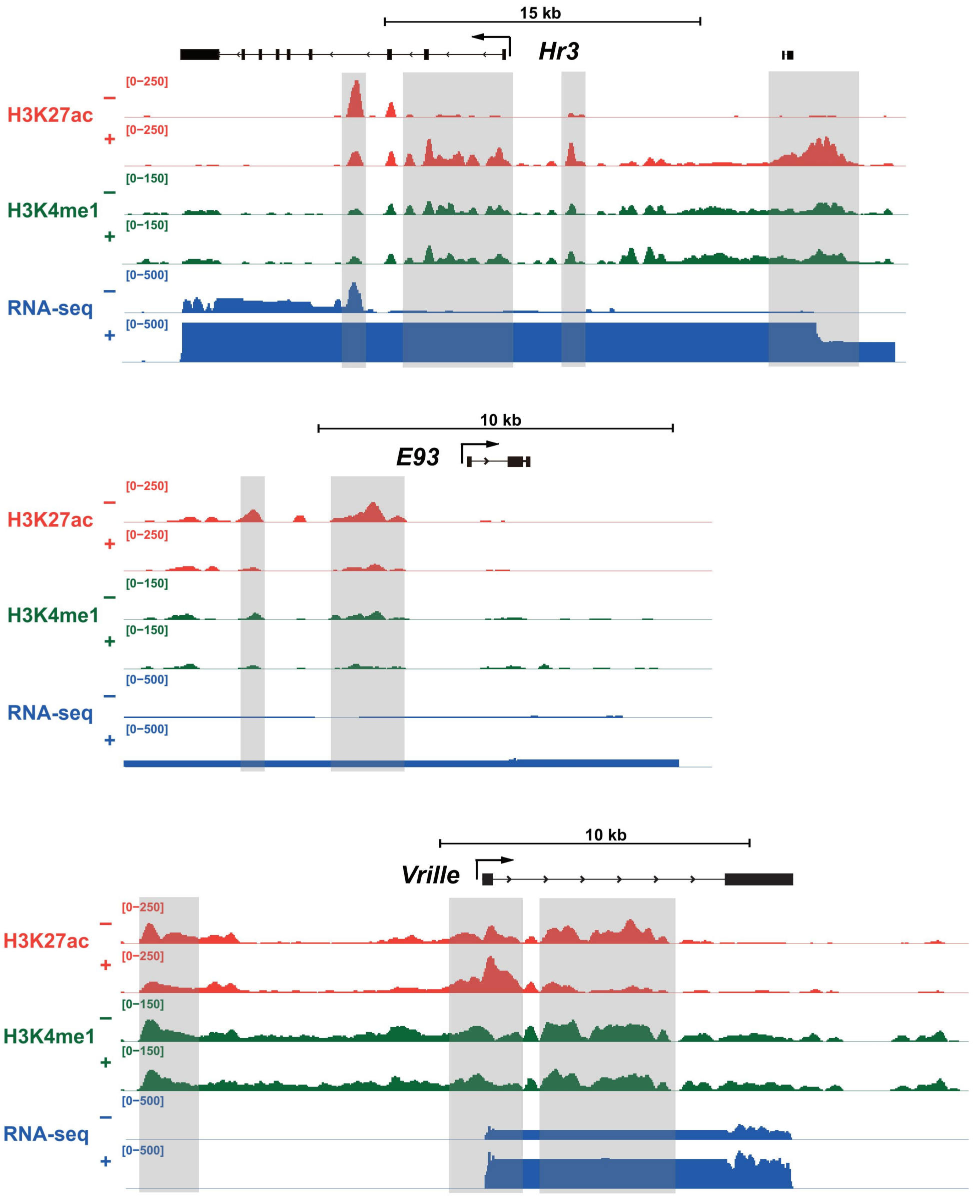

Supplementary Figure S1: IGV genome browser screenshots of ChIP-seq and RNA-seq tracks for the *Hr3*, *E93*, and *Vrille* gene loci. Grey shading highlights differential H3K27 acetylation regions. + and –

indicate the presence or absence of 20E treatment, respectively.

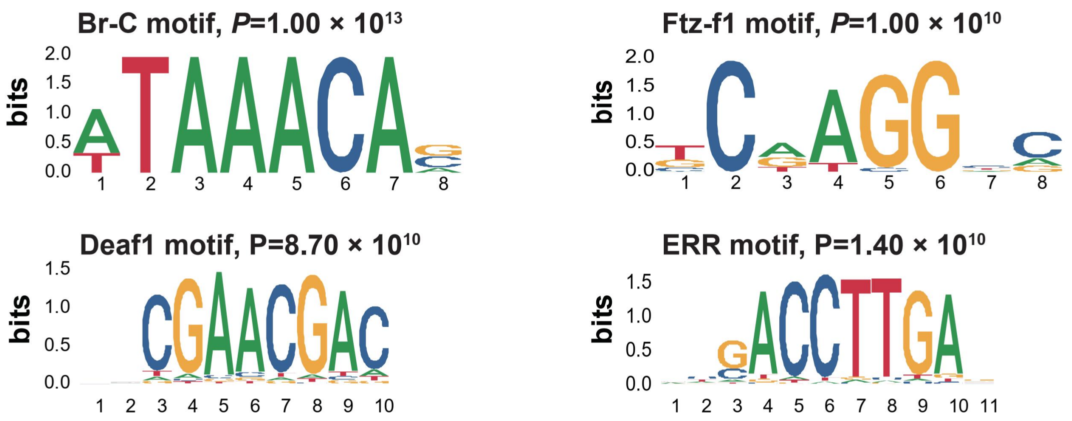

Supplementary Figure S2: *De novo* motif analysis of enhancers with increasing or decreasing H3K27 acetylation upon 20E treatment. The second enriched motif is shown.

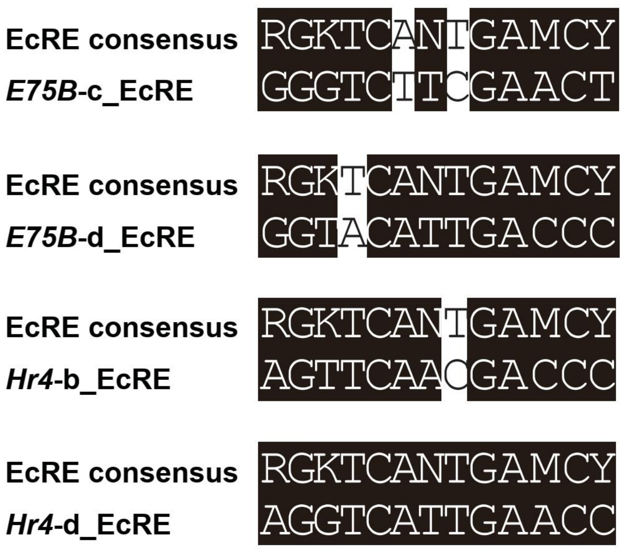

Supplementary Figure S3: The predicted EcRE in the enhancers of *Eip75B and Hr4* gene loci. Enhancers of *E75B* and *Hr4* gene loci as shown in Fig. 1C and Fig. 1D. The putative EcRE of enhancers were aligned with EcRE consensus sequences.

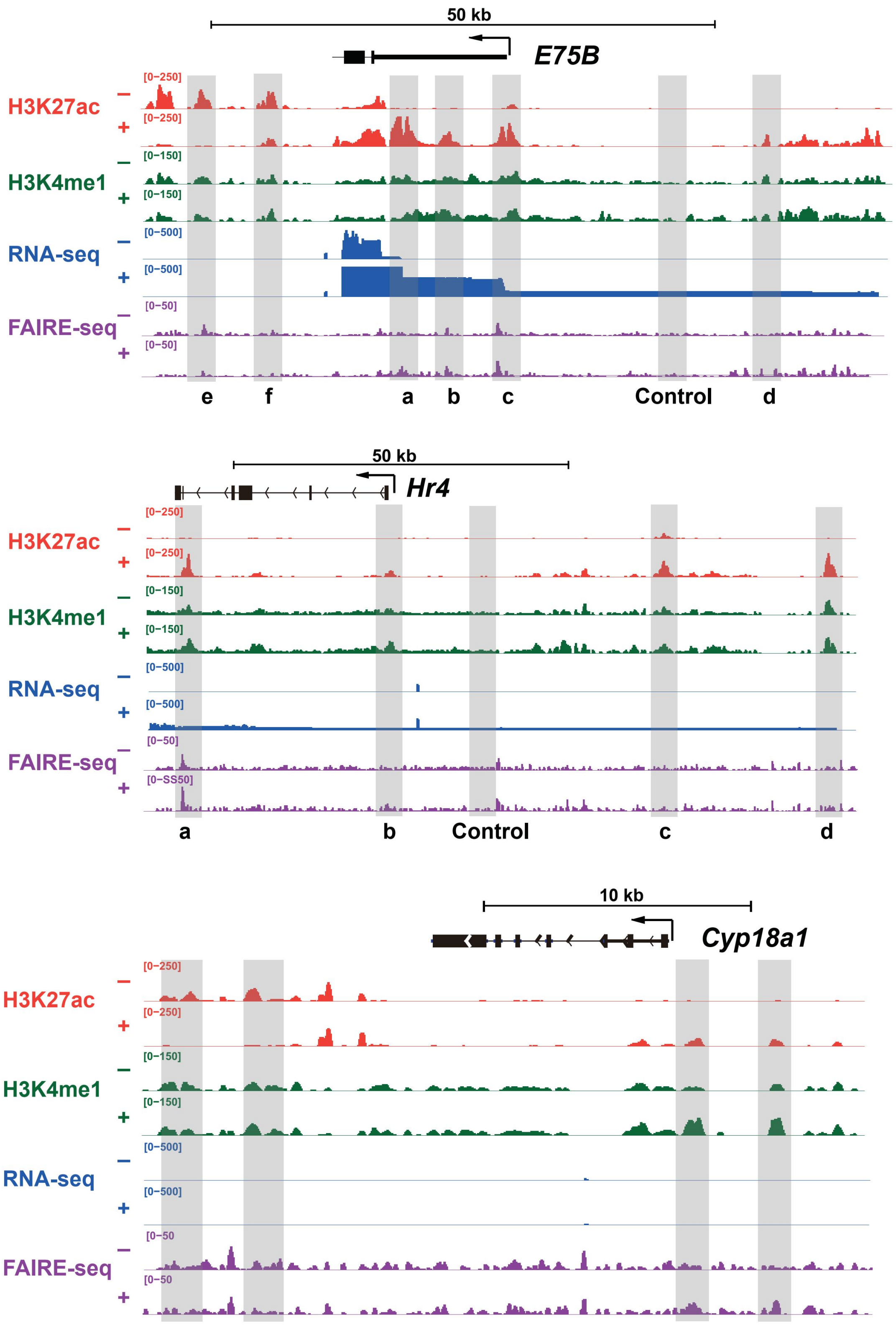

Supplementary Figure S4: IGV genome browser screenshots of ChIP-seq, RNA-seq, and FAIRE-seq tracks for *E75B and Hr4* gene loci. Orange and blue shading highlights enhancers that became open or closed, respectively, upon 20E treatment. + and − indicate the presence or absence of 20E treatment, respectively.

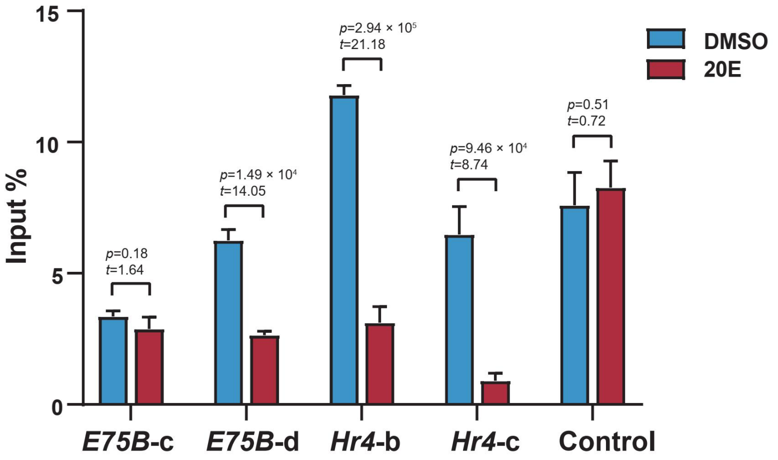

Supplementary Figure S5: ChIP-qPCR of H3K27me3 in DMSO- or 20E-treated BmE cells. DNA was quantified using primers designed to amplify the putative regulatory elements of *E75B* and *Hr4* gene loci as shown in Fig. 1C and Fig. 1E. Error bars: standard error of the mean (SEM) from three biological replicates measured in duplicate. Student's *t*-test (df=4).

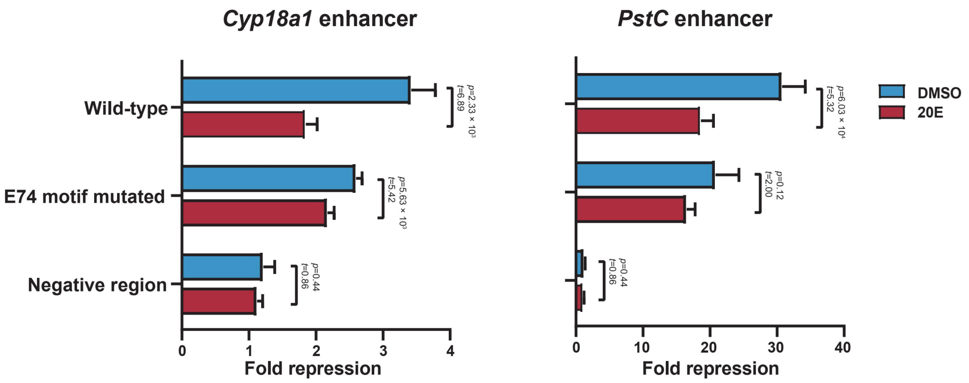

Supplementary Figure S6: Luciferase assays to detect changes in H3K27 acetylation, near *Cyp18a1* and *PstC* loci, of enhancers for which H3K27 acetylation decreased upon E20 treatment. The plot shows results for the wildtype sequence and an Eip74-motif mutant version, in which the enhancer does not contain an EcR motif. Negative region: sequence from the control region of *Hr4*, as in Fig. 1E. The DNA sequences are listed in Supplementary Table 2. Student's *t*-test (df=4).

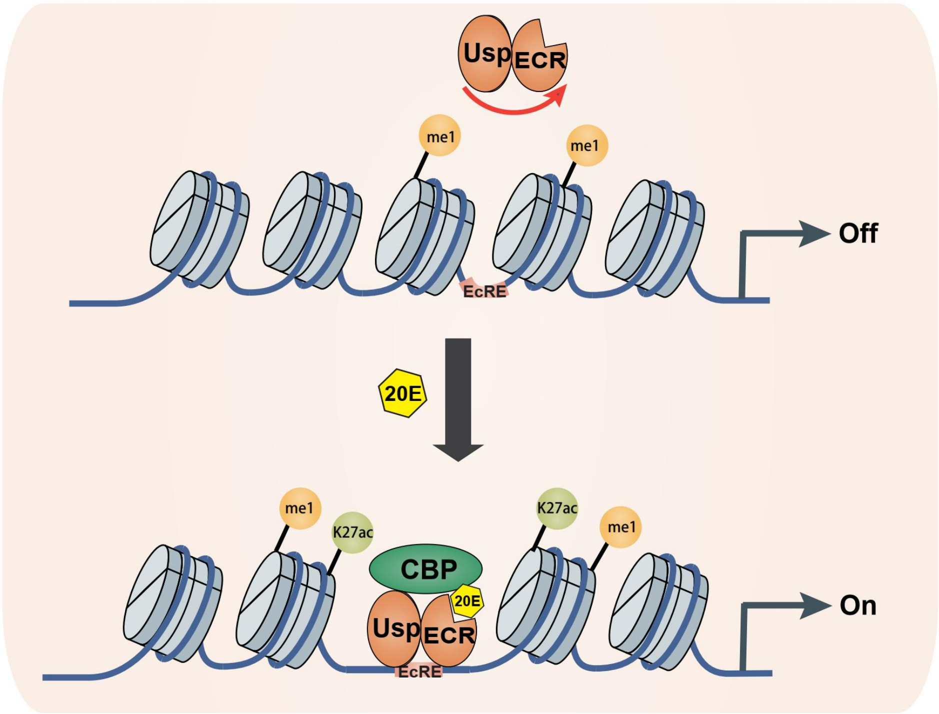

Supplementary Figure S7: Ecdysone facilitated ecdysone nuclear receptor (EcR) recruitment to chromatin. In the absence of a ligand, EcR cannot access the nucleosome-embedded EcRE. Binding of the ligand results in a conformational change in EcR, facilitating interaction with chromatin and the recruitment of the co-activator CBP, which in turn results in H3K27 acetylation and chromatin opening.

Supplementary tables:

Supplementary Table S1: Different classes of enhancers

Supplementary Table S2: Constructs Used for Luciferase Tests of Wild-Type and Mutant Enhancers, Related to Fig. 4B and Experimental Procedures. The mutated motifs are in bold, and changed nucleotides are in lowercase.

Supplementary Table S3: Primers Used for Amplification of Regions for Validations, Related to Experimental Procedures.
