# Supplementary material for: Transcriptional Activation of Ecdysone-Responsive Genes Requires H3K27 Acetylation at Enhancers
Source: Int J Mol Sci. 2022 Sep 16;23(18):10791. doi: 10.3390/ijms231810791 (PMC9502983; doi:10.3390/ijms231810791)
Supplement: Supplementary file 1 [file ijms-23-10791-s001.zip › ijms-1879961-supplementary/Supplementary Figure S3.pdf]

**EcRE consensus**	RGKTCANTGAMCY
***E75B*-c_EcRE**	GGGTCTTCGAACT
**EcRE consensus**	RGKTCANTGAMCY
***E75B*-d_EcRE**	GGTACATTGACCC
**EcRE consensus**	RGKTCANTGAMCY
***Hr4*-b_EcRE**	AGTTCAACGACCC
**EcRE consensus**	RGKTCANTGAMCY
***Hr4*-d_EcRE**	AGGTCATTGAACC
