# Supplementary figures and images for: Transcriptional Activation of Ecdysone-Responsive Genes Requires H3K27 Acetylation at Enhancers
Source: Int J Mol Sci. 2022 Sep 16;23(18):10791. doi: 10.3390/ijms231810791 (PMC9502983; doi:10.3390/ijms231810791)

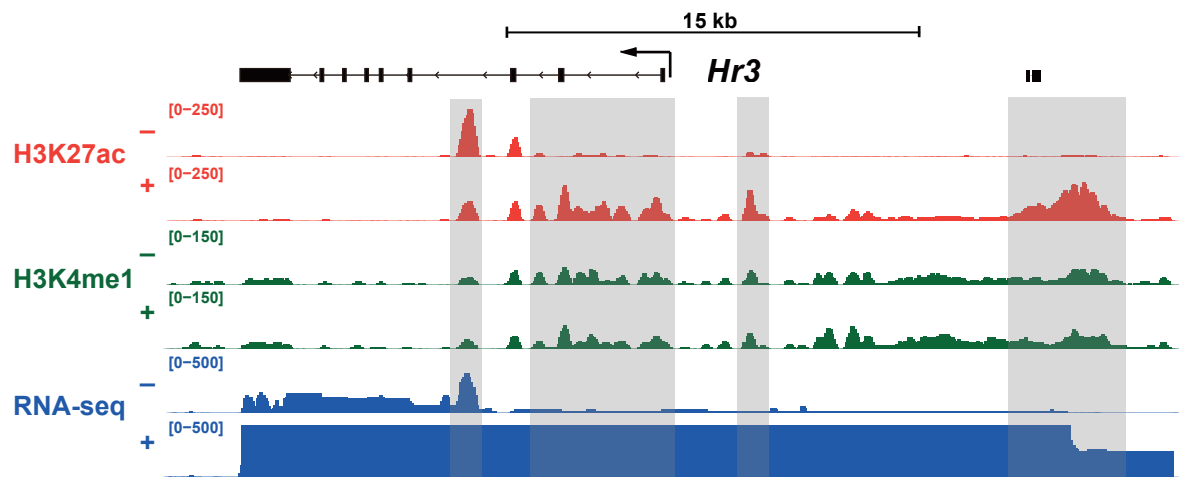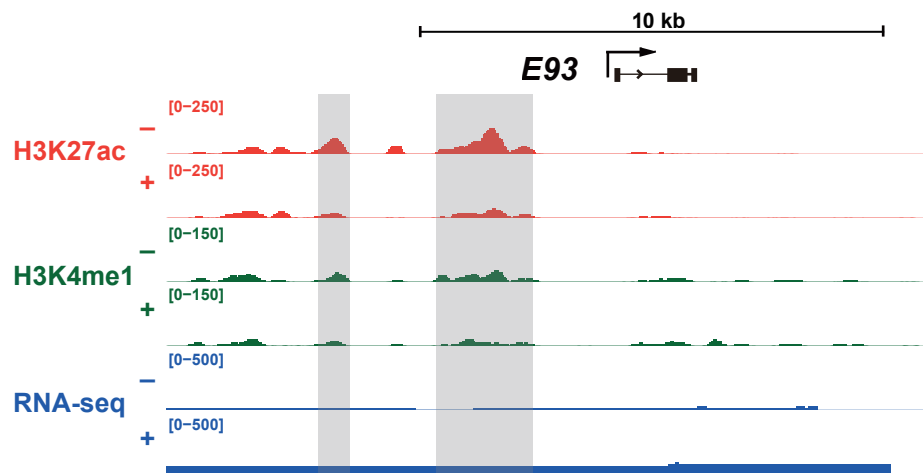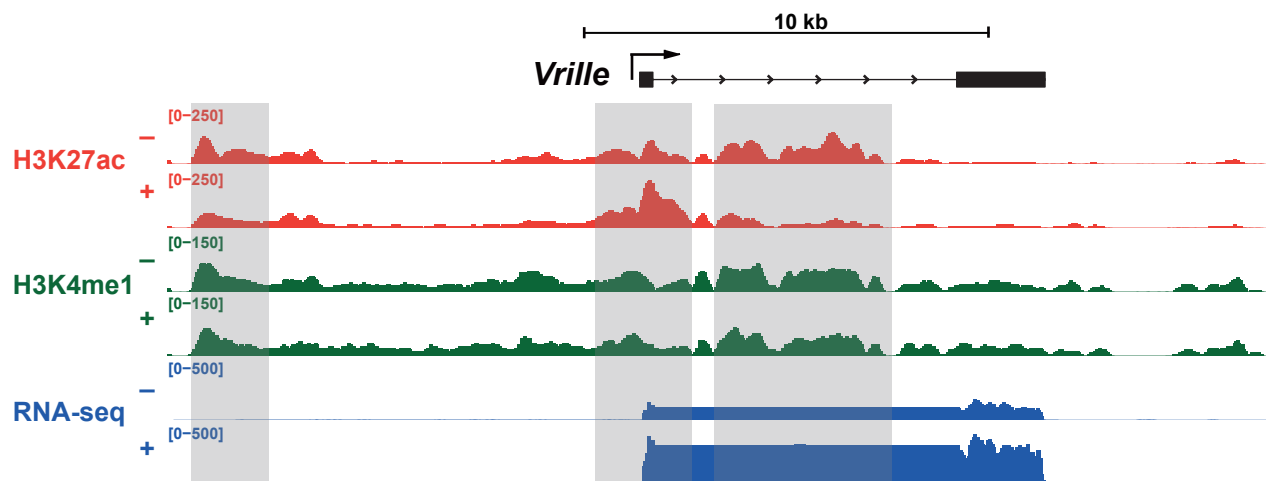

Supplement: Supplementary file 1 [file ijms-23-10791-s001.zip › ijms-1879961-supplementary/Supplementary Figure S1.pdf]

**Br-C motif,  $P=1.00 \times 10^{13}$**

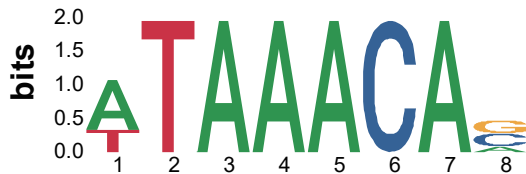

**Ftz-f1 motif,  $P=1.00 \times 10^{10}$**

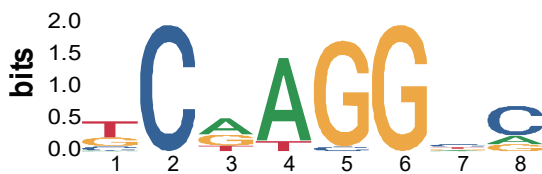

**Deaf1 motif,  $P=8.70 \times 10^{10}$**

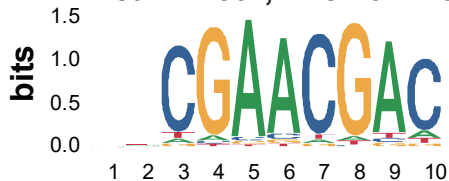

**ERR motif,  $P=1.40 \times 10^{10}$**

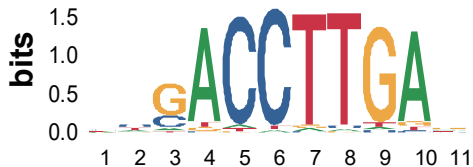

Supplement: Supplementary file 1 [file ijms-23-10791-s001.zip › ijms-1879961-supplementary/Supplementary Figure S2.pdf]

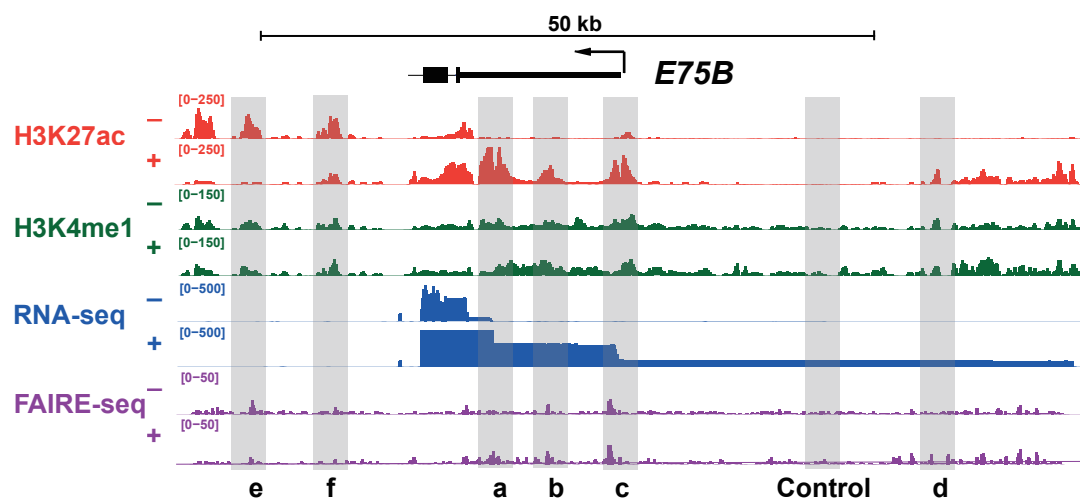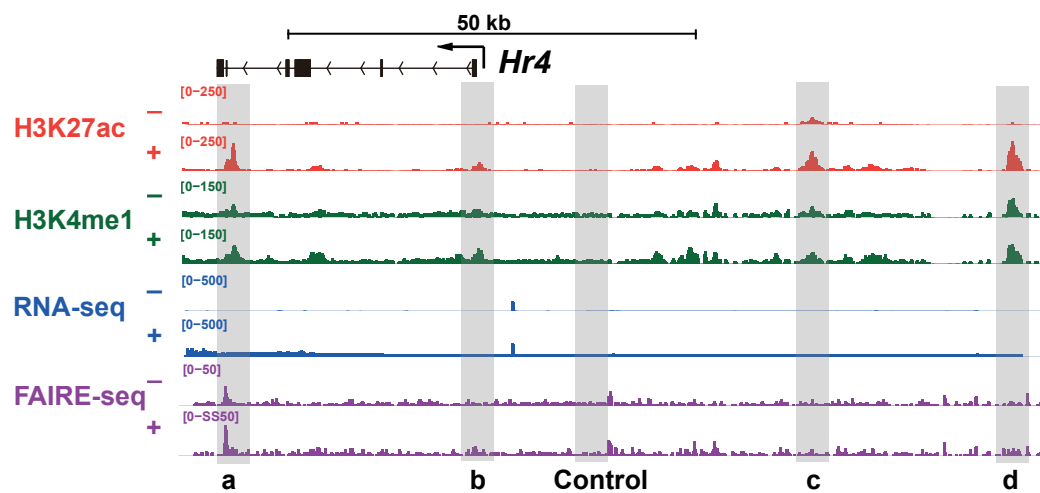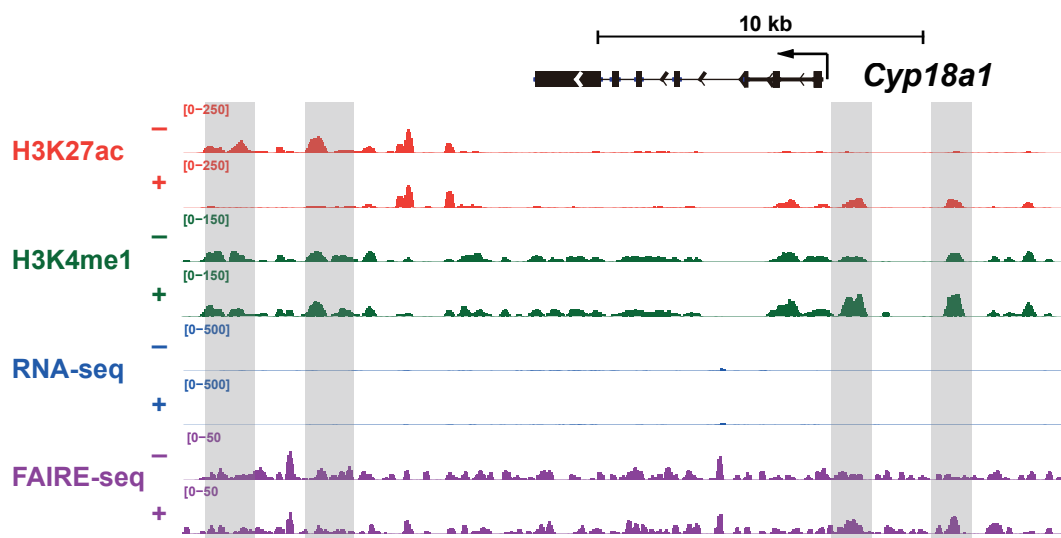

Supplement: Supplementary file 1 [file ijms-23-10791-s001.zip › ijms-1879961-supplementary/Supplementary Figure S4.pdf]

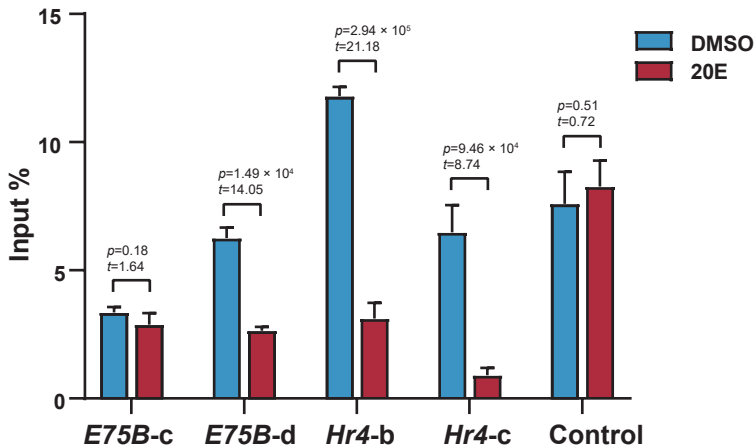

Supplement: Supplementary file 1 [file ijms-23-10791-s001.zip › ijms-1879961-supplementary/Supplementary Figure S5.pdf]

*Cyp18a1* enhancer

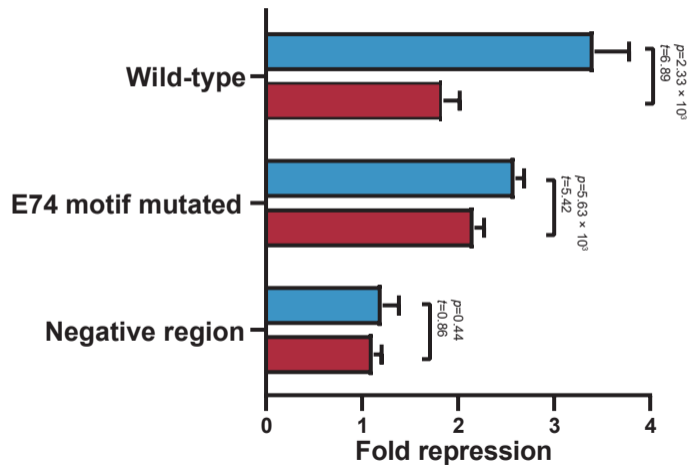

*PstC* enhancer

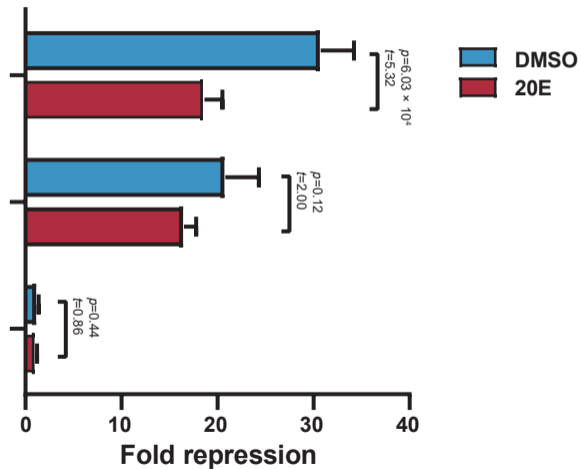

Supplement: Supplementary file 1 [file ijms-23-10791-s001.zip › ijms-1879961-supplementary/Supplementary Figure S6.pdf]

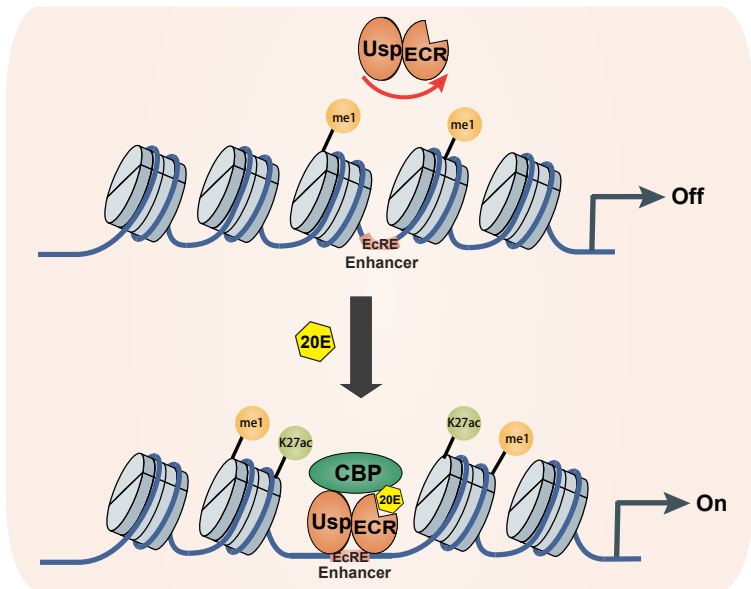

Supplement: Supplementary file 1 [file ijms-23-10791-s001.zip › ijms-1879961-supplementary/Supplementary Figure S7.pdf]
